# Mechanisms That Generate Resource Pulses in a Fluctuating Wetland

**DOI:** 10.1371/journal.pone.0158864

**Published:** 2016-07-22

**Authors:** Bryan A. Botson, Dale E. Gawlik, Joel C. Trexler

**Affiliations:** 1 Department of Biological Sciences, Florida Atlantic University, Boca Raton, Florida, United States of America; 2 Department of Biological Sciences, Florida International University, Miami, Florida, United States of America; University of Waikato (National Institute of Water and Atmospheric Research), NEW ZEALAND

## Abstract

Animals living in patchy environments may depend on resource pulses to meet the high energetic demands of breeding. We developed two primary *a priori* hypotheses to examine relationships between three categories of wading bird prey biomass and covariates hypothesized to affect the concentration of aquatic fauna, a pulsed resource for breeding wading bird populations during the dry season. The fish concentration hypothesis proposed that local-scale processes concentrate wet-season fish biomass into patches in the dry season, whereas the fish production hypothesis states that the amount of dry-season fish biomass reflects fish biomass production during the preceding wet season. We sampled prey in drying pools at 405 sites throughout the Florida Everglades between December and May from 2006–2010 to test these hypotheses. The models that explained variation in dry-season fish biomass included water-level recession rate, wet-season biomass, microtopography, submerged vegetation, and the interaction between wet-season biomass and recession rate. Crayfish (*Procambarus spp*.) biomass was positively associated with wet-season crayfish biomass, moderate water depth, dense submerged aquatic vegetation, thin flocculent layer and a short interval of time since the last dry-down. Grass shrimp (*Palaemonetes paludosus*) biomass increased with increasing rates of water level recession, supporting our impression that shrimp, like fish, form seasonal concentrations. Strong support for wet-season fish and crayfish biomass in the top models confirmed the importance of wet-season standing stock to concentrations of fish and crayfish the following dry season. Additionally, the importance of recession rate and microtopography showed that local scale abiotic factors transformed fish production into the high quality foraging patches on which apex predators depended.

## Introduction

When food is spatially and temporally variable, animals must track resources efficiently to match the costs of their feeding efforts to the energetic demands of their life history [1,2]. Reproduction is energetically costly, greatly elevating these demands, compelling foragers to target highly rewarding prey patches to sustain breeding [3–5]. A strategy employed by many organisms is to time breeding with resource pulses—infrequent, large magnitude, and short duration events of dramatically increased resource availability [6,7]. Resource pulses can occur intermittently, such as insect outbreaks [8,9], mast fruiting by trees [10–12], and irruptions of small mammal populations [13], or they can be seasonally recurrent events such as annual salmon spawning [14,15], seasonal inundation of river floodplains [16], and spawning of Pacific Herring (*Clupea pallasii*) [17]. Species living in environments where the spatial and temporal variability in food is integrally tied to recurrent pulses may evolve to completely rely on them [18].

Nesting wading birds (Pelecaniformes, Ciconiiformes), top predators in wetland ecosystems, are often limited by food [19–23] and may depend on ephemeral pulses of concentrated prey to sustain themselves during their breeding season [24–26]. In one large wetland, the Florida Everglades, wading birds are largely absent during the wet season, when water levels are deep, and prey are dispersed. During the dry season, large numbers of breeding wading birds come to exploit the resource pulses generated by receding water concentrating prey in shallow depressions [26,27]. Much is known about how birds respond to water level fluctuations [23,28] and which factors produce prey populations during times of high water [29]; however, little is known about factors that control resource pulses just as the marsh is drying and wading birds are using the resource.

Much of the evidence that wading birds are food-limited is based on the observed sensitivity of wading birds to hydrologic conditions, assumed to be reflective of food availability. This stems from evidence that populations of fish, the primary prey for wading birds, respond positively to increases in water levels [29–31] and negatively to drought [32–34]. There is not a clear relationship between crayfish and increases in water levels, but crayfish have been shown to respond positively following droughts due to a reduction in fish, which may release crayfish from predation [35]. However, droughts also can cause direct mortality to crayfish in short-hydroperiod wetlands [36]. Grass shrimp numbers are often low and slow to recover following drought [37], but their density and trophic position increases with time since dry-down [38,39]. These patterns led to the generalization that hydrologic conditions drive the production of aquatic prey organisms in wetlands [30,40,41]. However, the relative effect of particular hydrologic parameters on prey is not clear.

Production of prey is not the same as availability to wading birds because availability includes factors that affect the vulnerability of prey animals to being captured [26]. Moreover, the timing and magnitude of the response to hydrologic patterns differs strongly among prey species [37]. Gawlik [26] suggested that wading birds were responding to the components of prey availability that controlled vulnerability of prey to capture (e.g. vegetation) and the reorganization of prey into small dense patches (e.g. water depth), rather than to prey population size. While it is intuitive that fish production is a prerequisite for dense patches of prey, the relative effect of other ecosystem processes on generating seasonal pulses of concentrated prey for apex predators could be equally or more important, but are typically ignored. This study aimed to quantify the effect of key hydrological and habitat parameters on dry-season prey biomass, a pulsed resource that supports breeding wading bird populations.

**Fig 1:**
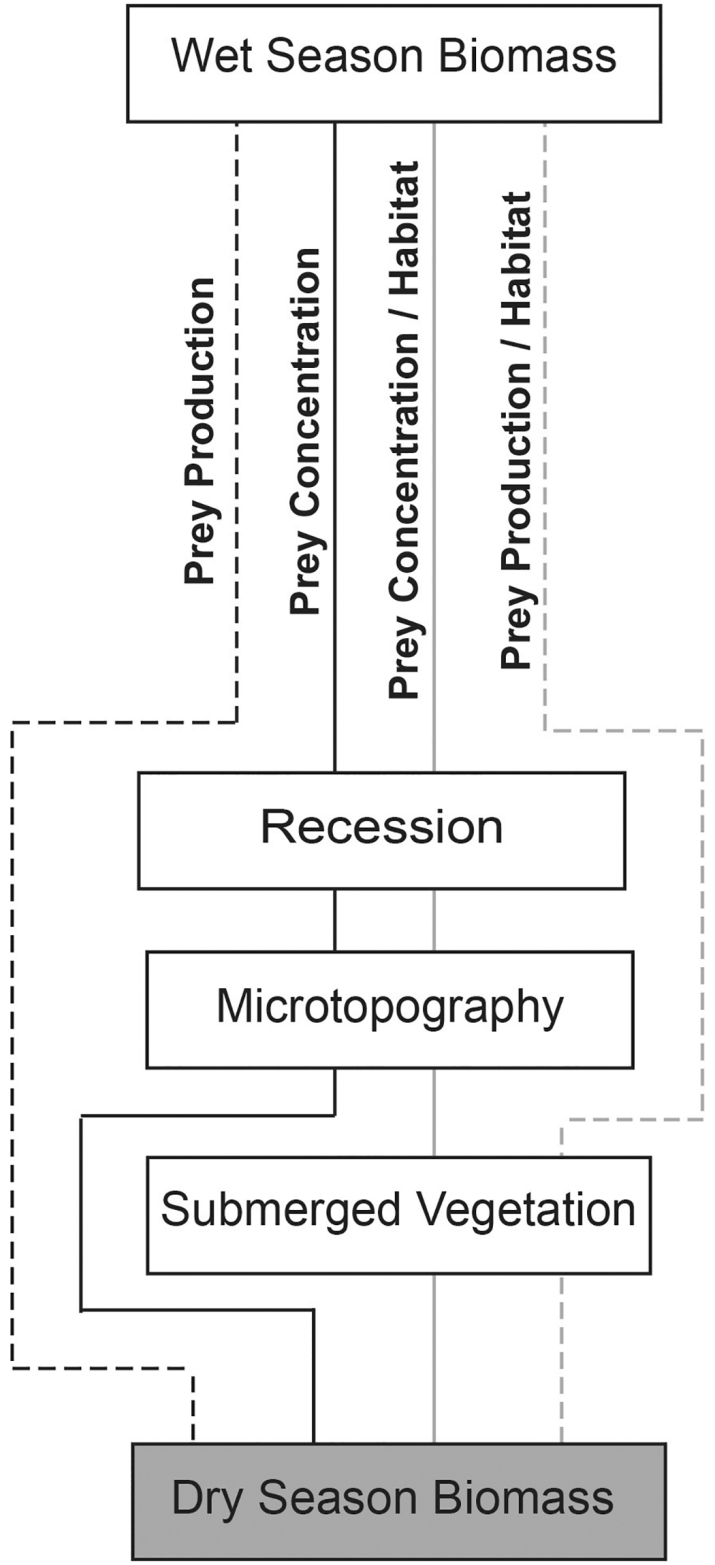
Conceptual model outlining hypotheses for factors effecting dry-season wading bird prey concentration in the Florida Everglades. Hypotheses are delineated by solid or dashed grey and black lines.

We tested *a priori* hypotheses about which factors were most important for generating high concentrations of dry-season fish, crayfish, and grass shrimp (Fig 1; S3 Appendix). These three taxon groups, which have different hydrological requirements, are the primary prey for wading birds, although prey preference differs among wading bird species [25,42,43]. Below we describe two core hypotheses, the “prey production” and “prey concentration”, which we tested to determine whether fish production during the wet season was sufficient to predict fish biomass during the dry season or whether physical factors that concentrate prey were also important. We also explore two additional hypotheses to determine the effect of habitat features on fish concentrations. Previous studies have indicated that recession and microtopography are important mechanisms for transforming wet-season fish populations into concentrated patches of fish biomass during the dry season [26,27,40]. As water levels recede, small changes in elevation form depressions that trap and concentrate fish. Thus, we proposed a “fish concentration hypothesis”, predicting that fish biomass would be highest at sites with high levels of wet-season fish biomass, high recession rates, and high microtopography (Fig 1). We also proposed a “fish concentration / habitat hypothesis” (Fig 1), predicting that fish biomass would be high where submerged vegetation was dense, as was seen in several studies [44–46]. We investigated two alternative “fish concentration hypotheses”, using days since dry-down or thickness of the flocculent matter (hereafter “floc”) as surrogates for wet-season fish biomass. Both floc and days since dry-down could be good predictors of wet-season prey biomass. Long periods of inundation increase time for growth and reproduction of fish populations [29], and fish and macroinvertebrate standing stocks are higher in habitats with enriched phosphorus [47,48], which accumulates in floc [49]. We also tested “fish production” and “fish production / habitat” hypotheses (Fig 1) as alternatives to the fish concentration hypotheses. These models exclude the local scale mechanisms that promote fish concentration and focus on the effect of wet-season fish standing stock on dry-season biomass.

Based on previous studies we hypothesized that crayfish biomass would increase with increased density of submerged vegetation and days since dry-down, but would decrease with increased fish biomass and water depth [35,36,38]. Because crayfish burrow when water levels drop, we expected that fast recession rates and microtopography would not result in high crayfish biomass. Since grass shrimp do not burrow, we hypothesized that grass shrimp biomass, like fish, would be positively correlated with recession rate and microtopography. Based on evidence that shrimp populations respond negatively to predation pressure by crayfish, but positively to density of submerged vegetation [38] and days since dry-down [39], we hypothesized that shrimp biomass would be highest at sites with high submerged vegetation, a long period of days since dry-down, and low crayfish biomass.

## Methods

### Study area

**Fig 2:**
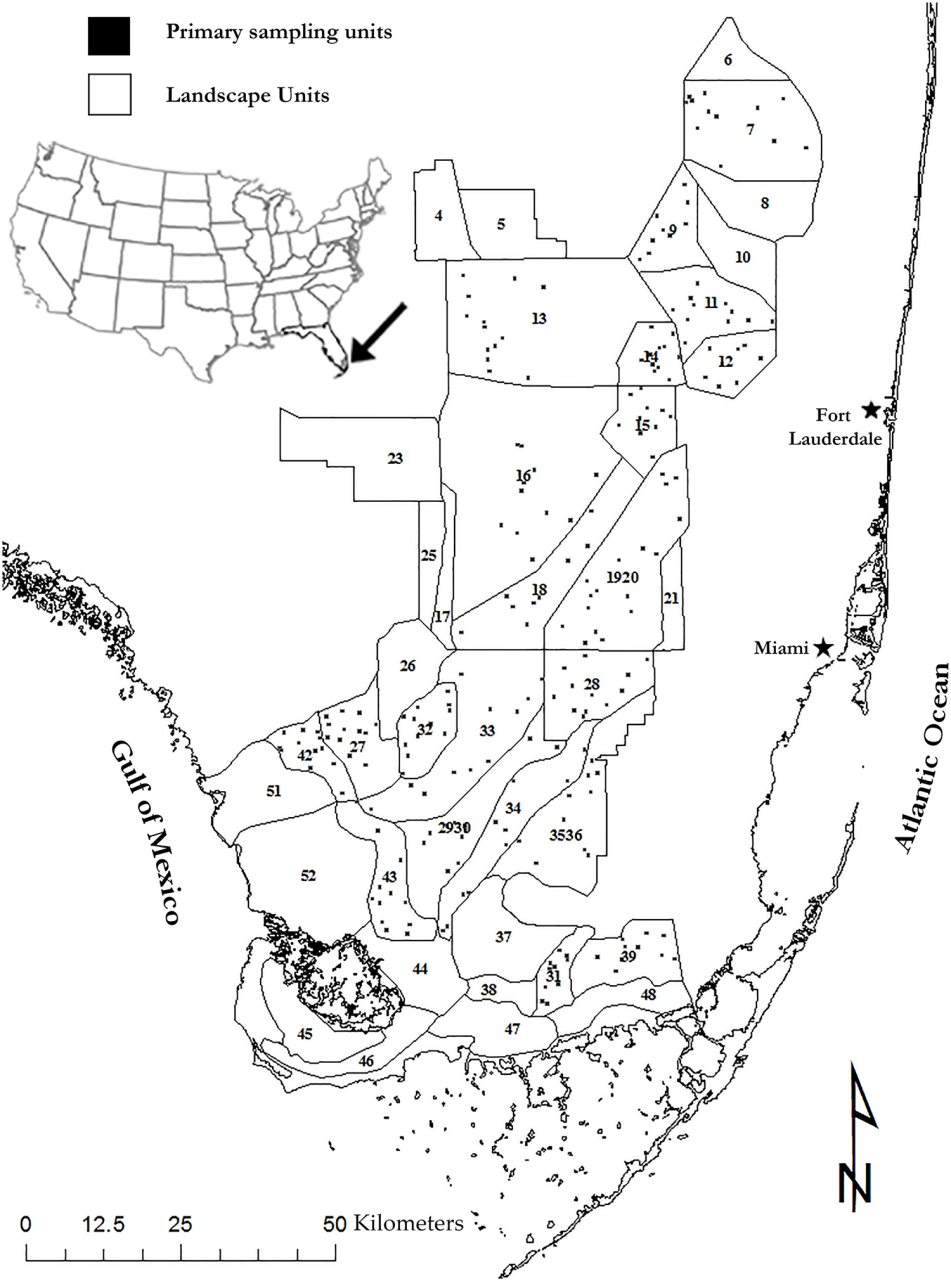
The Everglades of southern Florida and the set of landscape units from which we drew samples. Small squares indicate randomly located primary sampling units 500 m x 500 m in size. The bounding coordinates of the study site are: North 26.69, South 24.97, East -80.06, and West -81.53.

Our study region encompassed most of the freshwater portion of the Florida Everglades, about 7,000 km^2^ (Fig 2). This expansive freshwater marsh has a mosaic of habitats including sawgrass marshes, wet prairies, open-water sloughs, and tree island communities [50]. We sampled wading bird prey primarily in peat and marl wet prairies and open-water sloughs. Wet prairies with peat substrate occur in low elevation, deep regions of the central Everglades. Dominant plant species in these areas are spikerushes (*Eleocharis sp*.), beakrush (*Rhynchospora tracyi*), and maidencane (*Panicum hemitomon*) [50, 51]. Marl prairies occur at slightly higher elevations and have shorter hydroperiods than wet prairies, and are dominated by muhly grass (*Muhlenbergia* sp.) and sawgrass (*Cladium jamaicense*) [51]. Sloughs occur on the lowest elevations, have the longest hydroperiods, and are dominated by emergent macrophytes water lily (*Nymphea odorata*) and floating heart (*N*. *aquatica*), as well as submerged aquatic vegetation such as bladderwort (*Utricularia sp*.) [51]. Slightly higher than sloughs, sawgrass covered ridges form the slough edges and run parallel to the direction of water flow. Levees and canals divide the northern Everglades into five separate Water Conservation Areas. The southern Everglades includes Everglades National Park and Big Cypress National Preserve. Pronounced variation in seasonal rainfall creates distinct wet (May-October) and dry (October-May) seasons.

### Sampling design

We used a multi-stage sampling design [52] with landscape units (LSU), primary sampling units (PSU; Fig 2), sites within the PSUs, and 1-m^2^ throw-trap subsamples (TT) to quantify dry-season wading bird prey biomass from 2006 through 2010. A throw-trap is a 1-m^2^ box with mesh sides and an open top and bottom. A study on efficiency showed that this is an unbiased method for sampling fish in vegetated habitats with stem densities in the range of this study [53]. Landscape Units (Fig 2) were delineated primarily by hydroperiod and vegetation. Within each LSU, at least seven PSUs (500 m × 500 m) were established at random locations using ArcGIS 9.3 (ESRI Inc., Redlands, CA, USA). Within each PSU the locations of two random points were generated. The closest suitable habitat to the random point marked the TT site. Suitable habitat was habitat in which wading birds could forage, defined as an area with sparse to moderate vegetation with less than one-third of its surface covered with water. This criterion was based on knowledge from previous studies on the conditions targeted by foraging wading birds and how, when and under what conditions they aggregate across the Everglades landscape [26,54,55]. Within each site, aquatic fauna were sampled from two random TT (S1 Appendix).

### Site selection

We used the Everglades Depth Estimation Network (EDEN), field depth measurements, aerial site photos from previous years, and personal observations to identify PSUs with both surface water and exposed soil substrate, indicating that a PSU was probably at the target water depth. EDEN is a real-time hydrologic monitoring network that provides daily water depths at a spatial scale of 400 m x 400 m for most of the freshwater portion of the Everglades [56]. During each prey sampling event, we verified the suitability of habitat (shallow water and sparse vegetation) at a PSU thought to be at target water levels. If the PSU was at target water levels, we visited two random points within the PSU. We flew two to three east-west transects across the PSU to identify the closest suitable habitat to each point and to estimate the percentage of suitable habitat. The sampling team was dropped off by helicopter downstream from the closest suitable habitat to the random point to avoid disturbance. We selected the TT sites sequentially using random bearings and distances within suitable habitat, ensuring separation by at least 10 m.

### Sample collection

We measured vegetation structure, floc, and water depths in each of four quadrants of the throw-trap and then removed all vegetation within the trap to collect of aquatic fauna. We removed the aquatic fauna from the throw-trap by passing a 100-cm × 40-cm bar seine through the water column and floc until we had five consecutive sweeps with no fish or macroinvertebrates. We transferred captured fauna < 15 cm in length directly from the bar seine to jars containing a solution of water and MS 222, a rapid euthanizing agent. Larger fauna were identified, measured, and released. Once the trap was cleared, we stored all samples on ice until transfer to a solution of Prefer fixative in the laboratory. Approximately 1 week later, we filled sample jars with a 70% ethanol solution for permanent storage. We conducted all sampling with approval from the Florida Atlantic University Institutional Animal Care and Use Committee under Protocol A04-05 and the sampling protocol and all sampling methods were reviewed by the committee before we obtained a permit to conduct sampling.

We identified 99.9% of fish and 54% of crayfish to species. Only 4% of crayfish biomass was from unidentified crayfish greater than 2 cm total length, considered to be the minimum size for a wading bird prey item. Eight percent of the total fish, crayfish, and grass shrimp biomass (pooled across all years) was from prey items smaller than 2 cm total length. We weighed all individuals to the nearest 0.01 g, and measured standard length and total length for all fish and carapace length and total length for crayfish. We measured total length for invertebrates with irregular body shapes (e.g., shrimp). Biomass of fish, crayfish, and grass shrimp was calculated as the summed weight of all individuals within their respective taxonomic groups collected at a TT. Microtopography was characterized by measuring water depth every 1-m along a transect perpendicular to the direction of water flow; typically east-west in the northern Everglades and northwest-southeast in the southern Everglades. One 100-m transect was centered on the first TT at each site. When a transect reached a ridge, it was discontinued after three measurements (15 m) because ridges are not habitat for wading birds or their prey during the dry season; thus, some transects were less than 100 m.

Hydrological variables were calculated from daily water depths obtained from EDEN. Days since dry-down was calculated by counting the number of days since water depth in a cell was less than zero. EDEN water depths are derived from a single elevation at the center of a 400-m x 400-m cell so that when EDEN depth is zero, there may be portions of the cell with standing water. Because we targeted sites that exhibited conditions suitable for wading bird foraging (i.e., shallow), the water depth recorded by EDEN at a site on the day it was sampled was often less than zero. In these cases, we calculated an adjusted days since dry-down as the maximum number of days a cell had water depth greater than zero during the previous water year (June–May). Daily recession rate was calculated by subtracting the water depth in a cell on a given sample date from the water depth four weeks prior and dividing by 28 days. Positive recession rates denoted declining water levels whereas negative recession rates indicated water level had increased, termed here a “reversal”.

To account for microtopographical variation in a slough, we calculated a microtopography index based on water depth measurements at transects. The microtopography index was the difference between the maximum and mean water depth on a transect. Submerged vegetation structure was measured within throw-traps and characterized using the point-quarter method [57], calculating the distance from the center point of the throw-trap to the closest piece of submerged live or dead vegetation, in each of the four quadrants. This distance was inversely proportional to the density of vegetation. We also measured the thickness of the floc layer and water column (distance from water surface to top of floc layer) in each quadrant of the throw-trap. Data on biomass of fish, crayfish and shrimp from the preceding wet season were obtained from a companion study, following similar throw-trap methods.

### Statistical methods

We used the information theoretic approach to investigate competing models [58]. We developed *a priori* candidate models based on relevant literature and our current understanding of factors that affect fish, crayfish and grass shrimp concentrations (S2 Appendix). To identify which *a priori* models were most parsimonious, we employed Akaike's Information Criterion for small sample sizes (AICc). We computed ΔAIC_i_ values to determine separation between the best model and the other candidate models. We then calculated model probabilities (w_i_) to gather additional support for the models. We calculated a likelihood version of the correlation coefficient for each candidate model to assess model fit [59].

To assess the relative importance of each predictor variable in the candidate set, we summed Akaike weights (w_i_) for each model containing the variable. Additionally, we calculated model-averaged parameter estimates to examine the relative influence of an explanatory variable on the response variable [59]. To account for model selection uncertainty, we calculated the unconditional standard error and 95% confidence intervals of the parameter estimates. We plotted the model-averaged predicted values against the observed values to gauge how well the top models represented the data.

We constructed generalized linear mixed models with the procedure Proc Mixed (version 9.2; SAS Institute Inc., Cary, NC) to quantify relationships between each of three categories of wading bird prey biomass and the covariates hypothesized to be important. As part of the variable screening process, we tested for collinearity among explanatory variables with a correlation analysis, excluding terms where r > 0.7. Prey biomass was the mean biomass of fish, crayfish, and shrimp at a site. We log transformed the response variables to conform to assumptions of normality. We included LSU as a fixed effect in every model to account for spatial variation in prey biomass across the Everglades. We included year and PSU, nested within LSU, as random class variables in every model to account for spatial and temporal differences in prey biomass. We included a null model with only the parameters year, LSU and PSU, nested within LSU, to assess the worth of the candidate models in the set [59].

## Results

### Hydrological conditions

**Fig 3:**
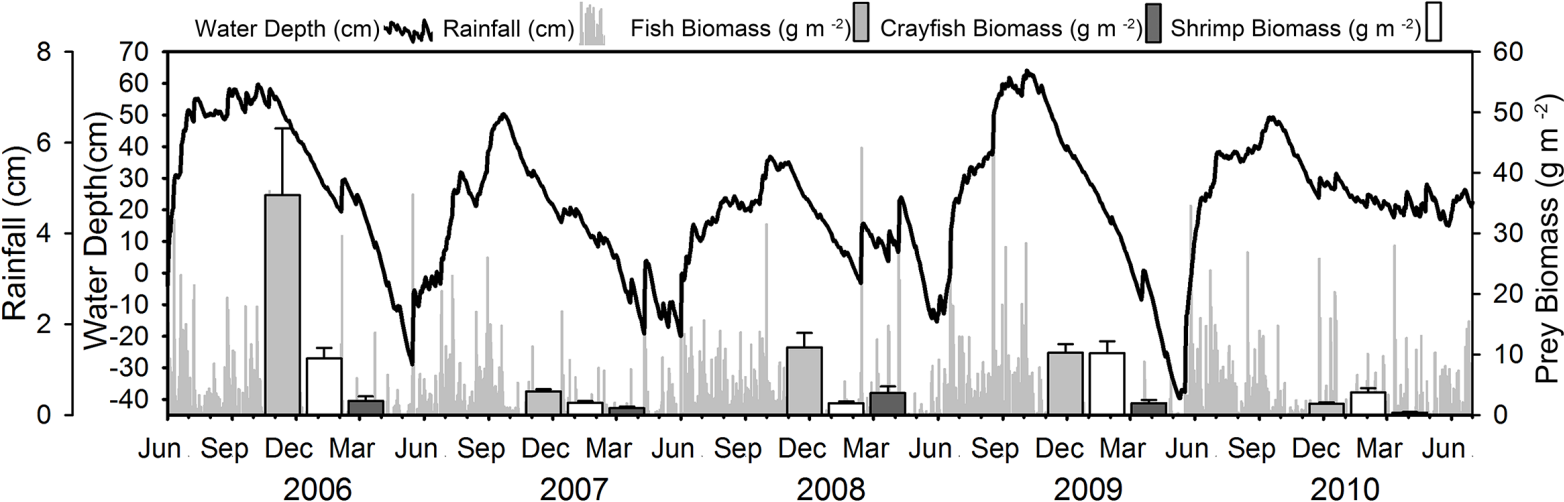
Mean water depth and rainfall for each day throughout the Florida Everglades from June 2006 to July 2010. Depth values represent the mean of 42,415 EDEN grid cells throughout most of the freshwater portion of the Everglades. Rainfall represents the mean of 18 rainfall gauges (NE4, NP202, NP203, P33, CR2, A13, NP206, NP62, P36, P34, TMC, NP205, BCA18, BCA19, BCA20, MDTS, S174, S20). Light grey, white, and dark grey bars represent fish, crayfish, and grass shrimp biomass (g m^-2^), respectively for 2006 to 2010 dry seasons.

Annual hydrologic conditions varied greatly during the five years of our study, as is common in subtropical wetlands (Fig 3). Water levels in 2006 were well above average at the start of the dry season and receded steadily throughout the season, unimpeded by major reversals in the drying pattern. Water levels in the 2007 and 2008 dry seasons were lower than average; however, 2008 was unique in that a series of rainfall events in mid-February considerably increased water levels system-wide (Fig 3), particularly in the northern Everglades, where they never receded to depths that could support wading bird foraging. Water levels in the 2009 dry season started just above average and then receded without much interruption (Fig 3). In contrast, water levels were higher during the 2010 dry season than any year in the past 10 years (Fig 3), and there was no seasonal dry-down.

### Temporal patterns

From 2006–2010, we collected 634 random TT samples at 405 sites and 211 PSUs throughout the Everglades. Additionally, we characterized microtopography along 405 transects. We collected 57,947 individual animals representing 34 taxa of aquatic fauna. Twelve species represented 99% of captured individuals (Table 1). When ranked by biomass, crayfish, Flagfish (*Jordanellae floridae*), Eastern Mosquitofish (*Gambusia holbrooki*), grass shrimp, Marsh Killifish (*Fundulus confluentus*), and Bluefin Killifish (*Lucania goodei*) were the six most abundant, accounting for 78% of total biomass. Pooled across all years, *P*. *alleni* was the most frequently captured crayfish species (Table 1).

**Table 1:** Species are presented in descending order of cumulative frequency representing 99% of individuals captured in throw-traps during 2006–2010 dry seasons.

Common name	Scientific Name	N	Biomass (g)	Length (mm)
Grass shrimp	*Palaemonetes paludosus*	19,302	1,350.63	19.41
Eastern Mosquitofish	*Gambusia holbrooki*	16,090	1955.43	16.54
Least Killifish	*Heterandria formosa*	5,581	265.56	12.79
Flagfish	*Jordanellae floridae*	5,133	2145.36	20.52
Bluefin Killifish	*Lucania goodei*	3,528	432.64	17.87
Crayfish	*Procambarus spp*.	1,528	929.78	11.84
Sailfin Molly	*Poecilia latipinna*	1,066	435.63	21.46
Everglades crayfish	*Procambarus alleni*	1,039	1,782.80	18.70
Marsh Killifish	*Fundulus confluentus*	859	575.56	28.96
Golden Topminnow	*Fundulus chrysotus*	839	395.14	25.35
Everglades Pygmy Sunfish	*Elassoma evergladei*	752	81.77	14.96
Slough crayfish	*Procambarus fallax*	694	984.81	18.11
Sheepshead Minnow	*Cyprinodon variegatus*	224	21.26	13.90
Total/Mean		56,635	11,356.37	18.49

N is the number of individuals captured. Mean total length is shown for grass shrimp. Mean carapace length is shown for crayfish and all fish species are shown in standard length. Biomass is reported as wet weight.

Mean total dry-season prey biomass (pooled across all samples) was highest in 2006 (58.09 g m^-2^ ± 15.36), and 2006 yielded the highest fish biomass (Table 2). Crayfish and shrimp biomasses were highest in 2009 (12.5 g m^-2^ ± 2.52) and 2008 (5.54 g m^-2^ ± 2.4), respectively, while overall prey biomass was intermediate in those years. Total dry-season prey biomass was lowest in 2010 (7.08 g m^-2^ ± 1.17), which had the lowest fish and shrimp biomass and the third lowest crayfish biomass (Table 2).

**Table 2:** Mean dry season prey biomass (measured as wet weight) and mean prey length (pooled across all sites) for 2005–2010 dry seasons.

YEAR	N	Fish		Crayfish		Shrimp	
		Biomass (g m^-2^)	Length (mm)	Biomass (g m^-2^)	Length (mm)	Biomass (g m^-2^)	Length (mm)
2006	51	43.62 ± 15.31	21.87 ± 0.08	12.03 ± 3.19	15.75 ± 0.21	2.43 ± 0.74	19.87 ± 0.09
2007	85	4.56 ± 0.57	16.93 ± 0.09	2.23 ± 0.43	12.43 ± 0.16	1.31 ± 0.33	18.36 ± 0.08
2008	78	15.59 ± 4.7	17.64 ± 0.07	2.86 ± 0.65	14.33 ± 0.33	5.54 ± 2.4	20.19 ± 0.05
2009	111	11.86 ± 2.04	17.23 ± 0.06	12.5 ± 2.52	16.41 ± 0.19	2.49 ± 0.84	18.98 ± 0.07
2010	80	1.99 ± 0.3	17.62 ± 0.21	4.62 ± 1.04	17.99 ± 0.42	0.47 ± 0.11	17.81 ± 0.25

Data shown as the mean ± 1 SE.

### Site characteristics

Water-level recession rates at sample sites were high in 2006 and 2009, moderate in 2007 and low in 2010 (Table 3). Microtopography index and throw-trap water depths were highest in 2006 and 2009 (Table 3), both years when a large portion of the landscape dried. Floc thickness at sites was high in 2009, moderate in 2007 and 2008, and low in 2006 (Table 3). Distance to submerged vegetation was much higher in 2006 (Table 3) than other years, indicating low density of submerged vegetation.

**Table 3:** Mean habitat variables and wet season prey biomass from 2006–2010.

Year	N	Recession rate (cm/day)	Micro-topography (cm)	Submerged vegetation (cm)	Floc thickness (cm)	Wet Season Biomass (g m^-2^)			
						N	Crayfish	Fish	Shrimp
2006	51	0.51 ± 0.04	22 ± 3.1	32 ± 8.8	6 ± 0.8	16	3.12 ± 0.68	1.55 ± 0.27	0.24 ± 0.09
2007	85	0.30 ± 0.02	13 ± 0.9	16 ± 3.6	7 ± 0.5	30	1.83 ± 0.40	1.43 ± 0.23	0.29 ± 0.08
2008	78	0.25 ± 0.06	14 ± 1.2	10 ± 1.9	8 ± 0.5	20	2.41 ± 0.38	2.21 ± 0.58	0.40 ± 0.09
2009	111	0.47 ± 0.02	22 ± 1.9	13 ± 2.0	10 ± 0.7	37	3.76 ± 0.81	2.12 ± 0.21	0.53 ± 0.09
2010	80	0.16 ± 0.02	13 ± 0.9	6 ± 0.6	5 ± 0.3	18	6.06 ± 1.39	1.19 ± 0.42	0.08 ± 0.03

Means are pooled across all sites. Habitat variables were recorded during the dry season, while wet season biomass was recorded during the previous wet season at sites near the dry season sites. Data shown as the mean ± 1 SE.

### Factors affecting wading bird prey biomass

#### Fish biomass

**Fig 4:**
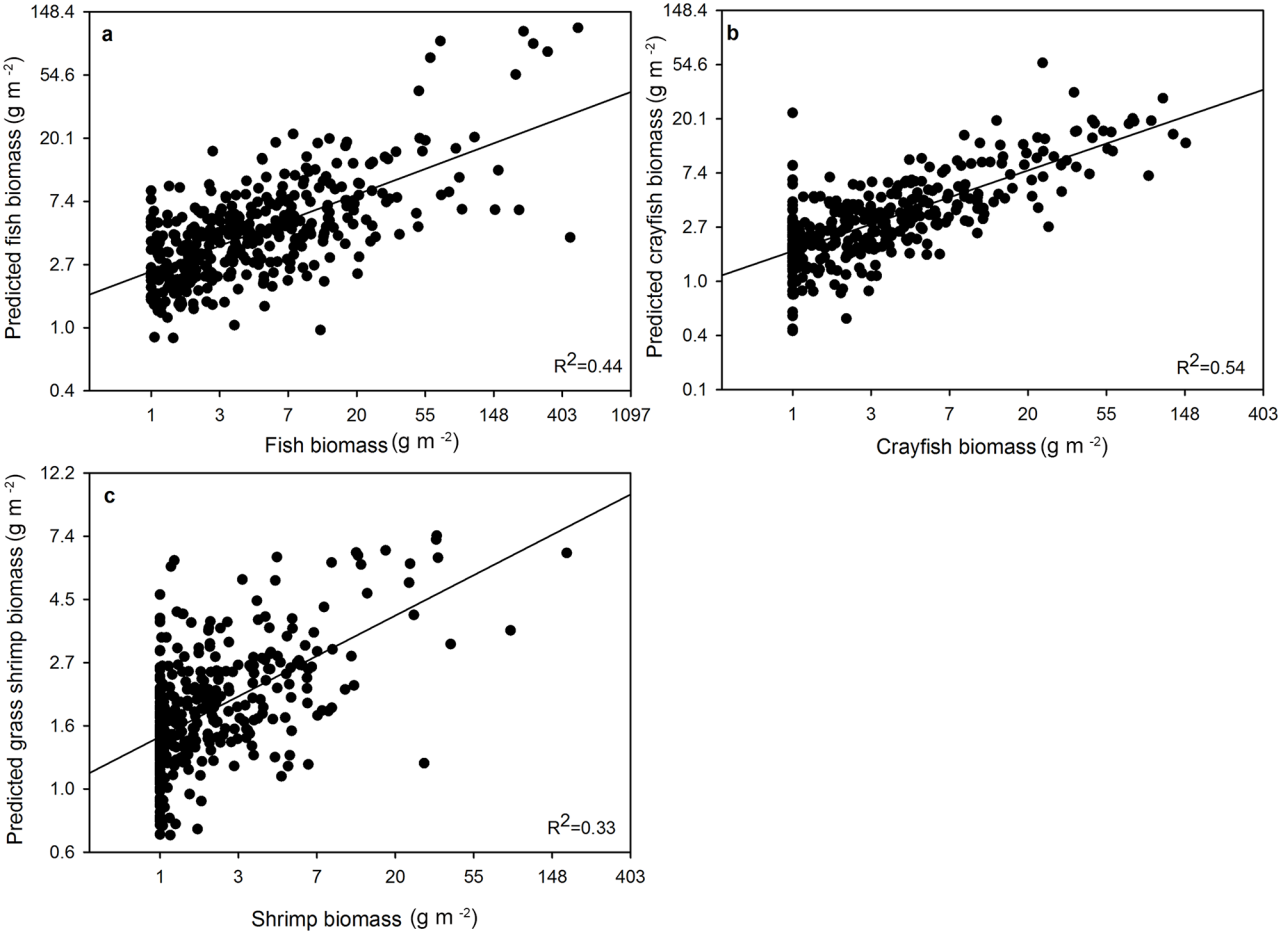
Model-averaged predicted values from models with (ΔAICc < 7) plotted against observed values. (a) fish biomass (gm^-2^), (b) crayfish biomass (g m^-2^), and (c) grass shrimp biomass (g m^-2^). All panels were plotted on log scale.

**Fig 5:**
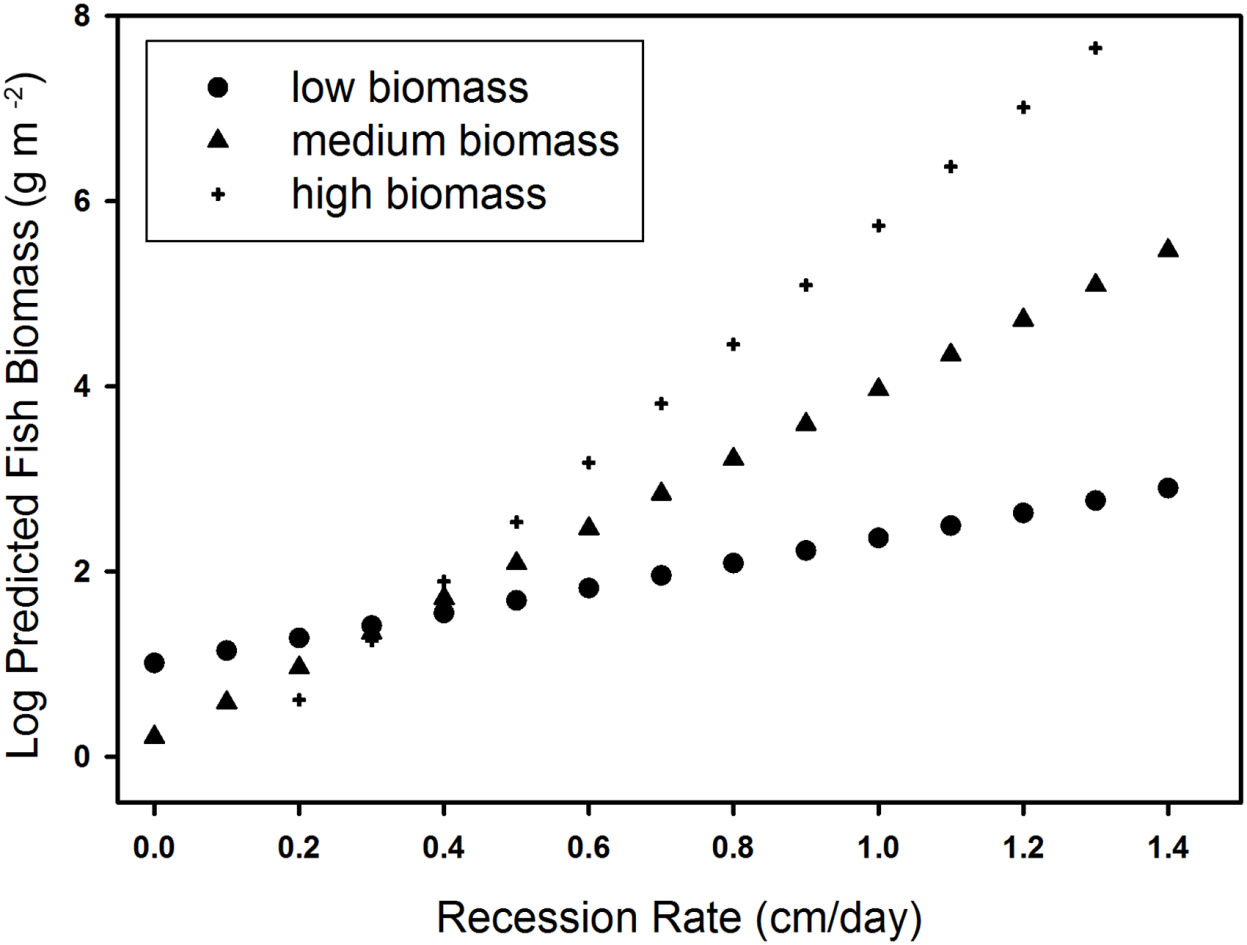
The relationship between predicted dry-season fish biomass (y-axis) and recession rate (x-axis). Low (circles), medium (triangle) and high (cross) levels of wet season biomass.

The model with the most support for explaining variation in dry-season fish biomass (w_i_ = 0.75; Table 4) included the terms recession, wet-season fish biomass, microtopography index, throw-trap submerged vegetation, and the interaction between recession and wet-season biomass. The second best model (w_i_ = 0.18; Table 4) contained the same parameters as the best model, but without the interaction term. These two models, representing the fish concentration / habitat hypothesis (Fig 1), accounted for 98% of the Akaike weight. The third best model (w_i_ = 0.07) was the global model, which contained three terms in addition to those in the 2^nd^ best model. It was within 6 AIC units of the two top models with a similar log-likelihood value, indicating that the additional parameters received little to no support [58,60]. The remaining models: fish concentration hypothesis without vegetation, and the alternative fish concentration hypothesis with days since dry-down or floc as a proxy for wet-season fish biomass, received almost no support (ΔAIC > 20). Model-averaged predicted values plotted against expected values showed a strong predictive relationship (Fig 4). There was a high positive effect of recession rate (1.1 cm/day ± 0.40) on dry-season prey biomass, indicating that increased rates of recession produced elevated fish biomass (Table 5). An increase in recession rate from 0.2 cm/day to 0.6 cm/day increased fish biomass by 55%. The positive coefficients for submerged vegetation distance (0.01 cm ± 0.004) and microtopography (0.01 cm ± 0.003) indicated that increased microtopography increased fish biomass, and that fish biomass was higher in areas with sparse submerged vegetation (Table 5). The interaction term for recession × wet-season fish biomass was positive, indicating that predicted fish biomass increased with increasing recession and wet-season biomass (Fig 5). However, fish biomass increased more rapidly with increases in the rate of recession (Fig 5) than to wet- season fish biomass.

**Table 4:** Results of generalized linear mixed-effects models of factors affecting fish, crayfish and grass shrimp biomass in the Florida Everglades, USA.

Model	-2 Loglike	k	AICc	ΔAIC	w_i_	R^2^
Fish Models						
- FISHBIOWET +MCRIND +REC +SUBMGVEG +FISHBIOWET*REC	1085.84	24	1137.17	0.00	0.75	0.3171
-FISHBIOWET +MCRIND +REC +SUBMGVEG	1090.94	23	1140.00	2.83	0.18	0.3073
GLOBAL	1083.73	27	1141.96	4.79	0.07	0.3211
	:	:	:	:	:	:
NULL	1224.11	19	1264.08	126.91	0.00	0.0000
Crayfish Models						
GLOBAL	972.52	27	1030.76	0.00	1.00	0.3824
	:	:	:	:	:	:
NULL	1146.08	19	1186.05	155.29	0.00	0.0000
Shrimp Models						
GLOBAL	804.54	25	858.17	0.00	0.71	0.2758
+ CRAYBIODRY +GRSHBIOWET +REC +MCRIND SUBMGVEG	810.86	23	859.92	1.75	0.29	0.2621
	:	:	:	:	:	:
NULL	915.39	18	953.16	95.00	0.00	0.0000

Only models with ΔAICc < 7 and null models are shown. We show -2 log likelihood value (-2 Loglike), number of parameters (k), AIC values, differences in AIC between best model and each candidate model (ΔAIC), AIC weights (**w**_**i**_) and the likelihood coefficient of determination (R^2^). FISHBIOWET, wet-season fish biomass; MCRIND, microtopography index; REC, recession rate; SUBMGVEG, submerged vegetation; CRAYBIODRY, dry-season crayfish biomass; GRSHBIOWET, wet-season shrimp biomass.

**Table 5:** Model-averaged parameters of factors affecting the concentration of fish, crayfish, and grass shrimp biomass.

Parameter^a^	β	LCL	UCL	Σw_i_
Fish models				
INTERCEPT	1.089	0.549	1.629	1.00
MCRIND	0.010	0.003	0.017	1.00
REC	1.109	0.335	1.883	1.00
FISHBIOWET	-0.080	-0.235	0.075	1.00
SUBMGVEG	0.008	0.004	0.012	1.00
FISHBIOWET×REC	0.241	0.022	0.460	0.82
Crayfish models				
INTERCEPT	0.631	0.164	1.099	1.00
FISHBIODRY	0.080	-0.006	0.167	1.00
DEPTH	0.058	0.043	0.073	1.00
CRAYBIOWET	0.037	0.012	0.062	1.00
SUBMGVEG	-0.009	-0.012	-0.005	1.00
DSD	-0.001	-0.002	-0.001	1.00
FLOC	-0.076	-0.101	-0.051	1.00
REC	0.704	0.295	1.113	1.00
MCRIND	-0.002	-0.009	0.005	1.00
Grass shrimp models				
INTERCEPT	-0.057	-0.362	0.247	1.00
MCRIND	0.004	-0.002	0.009	1.00
REC	0.733	0.416	1.049	1.00
GRSHBIOWET	0.080	-0.155	0.315	1.00
SUBMGVEG	0.000	-0.003	0.003	1.00
CRAYBIODRY	0.181	0.109	0.254	1.00
DSD	0.000	0.000	0.001	0.71
FLOC	-0.011	-0.025	0.004	0.71

Model-averaged parameter estimates (β), 95% confidence limits (LCL, UCL) and variable importance values (Σw_i_) for models (ΔAICc < 7) of factors affecting the concentration of fish, crayfish, and grass shrimp biomass. MCRIND,microtopography index; REC, recession rate; FISHBIOWET, wet-season fish biomass; SUBMGVEG, submerged vegetation; FISHBIODRY,dry season fish biomass; CRAYBIOWET,wet-season crayfish biomass; DSD,days since dry-down; FLOC, floc thickness; GRSHBIOWET, wet-season shrimp biomass; CRAYBIODRY, dry-season crayfish biomass.

#### Crayfish biomass

The global model (w_i_ = 0.99; Table 4) had substantially more support for explaining variation in dry-season crayfish biomass, and all other models had ΔAIC > 2. Model-averaging revealed that the most important parameters for explaining variation in dry-season crayfish biomass included wet-season crayfish biomass, recession rate, throw-trap submerged vegetation, floc thickness, days since dry-down, and throw-trap water depth (Table 5). Model-averaged predicted values plotted against expected values showed a strong predictive relationship (Fig 4). Dry-season crayfish biomass was positively associated with wet-season crayfish biomass, throw-trap water depth, and recession rate. An increase in recession rate from 0.2 cm/day to 0.6 cm/day increased dry-season crayfish biomass by only 20%. Crayfish biomass was negatively associated with floc thickness, indicating more crayfish biomass at sites with thinner floc. The negative effect of distance to submerged vegetation distance revealed that crayfish were more common in heavily vegetated areas than open areas. Predicted crayfish biomass doubled with a 55-cm decrease in distance to submerged vegetation and dropped by half with an 8-cm increase in floc thickness.

#### Shrimp biomass

The global model (w_i_ = 0.71; Table 4) had the most support for explaining variation in dry-season shrimp biomass; however, dropping floc had no impact on the model quality (ΔAIC < 2). Model averaging showed that recession rate and crayfish biomass were the only variables that had parameter estimates with confidence limits that did not overlap zero. There was a positive effect of recession rate on shrimp biomass, indicating that increases in the rate of recession increased dry-season shrimp biomass (Table 5). There was also a positive association of dry-season crayfish biomass, indicating that shrimp were more common in areas where crayfish were also abundant (Table 5).

## Discussion

Differences in factors that affected biomass of fish, crayfish, and grass shrimp were likely tied to their respective life history strategies. These patterns emerged even though fish and crayfish were multi-species groupings with known inter-specific differences in life histories. Fish and shrimp biomass had strong positive responses to recession rate, while crayfish showed a weak response, demonstrating that the mobile fish and shrimp concentrated as the marsh dried. Only fish biomass responded to microtopographical variation, indicating that fish seek out local depressions that serve as temporary refuges as the marsh dries. The strong support for wet-season fish and crayfish biomass in the top models confirmed the importance of wet-season standing stock to concentrations of fish and crayfish in the following dry season. Fish and crayfish showed opposite responses to density of submerged vegetation, likely due to differences in how each species responded to a drying marsh.

### Fish biomass

Both recession rate [26,61,62] and microtopography [26,40,63] were associated with high-quality foraging patches for wading birds in the Everglades. Additionally, we confirmed the importance of wet-season standing stock to the concentration of fish the following dry season and showed that the density of submerged vegetation also affected fish concentrations. Our findings supported the fish concentration \ habitat hypothesis, demonstrating that high fish concentrations are not solely a function of prey production, but that facilitating mechanisms such as recession and microtopography are required to increase fish biomass well above the giving-up-density threshold for wading birds [26]. Microtopographical relief creates shallow depressions that allow fish to concentrate before the marsh dries completely [26]. The local concentration hypothesis proposed by Trexler et al. [40] was supported by evidence that fish biomass was positively associated with variation in microtopography at the patch scale, likely concentrating in local depressions rather than travelling to seek out deep water refuges. Receding water distributes fish and macroinvertebrates into these depressions [26,27]. The rate of recession also affected the quantity of fish biomass concentrated within any given patch (Fig 5). The drying process is a characteristic of most wetlands, and there are examples from other systems of wading birds relying on seasonal recession to concentrate prey. For example, Wood Storks (*Mycteria americana*) in the southern Llanos of Venezuela preferred to forage in ponds and lagoons with receding water and high concentrations of fish during the dry season [25]. Also, Little Egrets (*Egretta Garzetta*) in the Camargue of southern France fed mainly in temporary marshes that dried out each summer [24], whereas heron and egret densities increased with decreasing water levels in coastal wetlands in Ghana [64]. The process of prey production during the wet season generating high quality prey patches during the following dry season was poorly understood prior to this study, although it was assumed to be important. The strong empirical support for wet-season fish biomass in the top models confirmed its importance, but also highlighted the value of the other factors that make prey available to wading birds. In many wetlands, seasonal water level recession may be the primary mechanism for creating recurrent resource pulses for breeding wading birds, and exceptional pulses occur when high fish production is followed by high rates of receding water.

The negative association of fish with submerged vegetation was contrary to our expectation. Fish generally are positively correlated with density of submerged aquatic vegetation [44–46], because vegetation reduces the risk of predation from aquatic predators, including wading birds [65–68]. Our study was done at shallow and nearly dry sites, increasing risk of predation from terrestrial predators such as birds. Indeed, some wading bird species prefer sites with moderate amounts of vegetation over sites with no vegetation [69–73]. This pattern suggests that, as water levels drop, risk of desiccation overrides risk-sensitive behavior to aquatic predators that is prevalent when water level is relatively high. As water levels recede in a drying marsh, an increasing proportion of the water volume is taken up by the vegetation. If fish do not seek out deep areas, with typically less vegetation [74], the vegetation can become an impediment, creating isolated pockets of water in what was otherwise a contiguous pool. Under these conditions, we (DEG pers. obs) observed a golden topminnow jump from a roughly 10-cm diameter pocket of water to the surface of what had become a surrounding mat of vegetation, and flip repeatedly, traveling meters, until it encountered another isolated pocket of water. Given the high risk of desiccation associated with movement above the water surface during mid-day, it was reasonable to conclude that movement through the vegetation by fish at this point was impossible.

The lack of support for days since dry-down was surprising because long periods of inundation foster prey production increasing the size and abundance of fish [29]. There is also evidence that fish species respond individualistically following a dry-down. Flagfish and Marsh Killifish rebound quickly following drought through either dispersal or rapid reproduction, whereas Bluefin Killifish, Least Killifish, and Golden Topminnow recover more slowly. Eastern Mosquitofish showed no clear response to a dry-down [63,75,76]. Differential behaviors of the species of fish could mask the overall effect of days since dry-down on dry-season fish biomass. The lack of support for floc could indicate that the effect of floc is overwhelmed by other factors influencing the concentration of fish, or that floc may not be a reliable indicator of nutrients or hydroperiod in a drying marsh.

### Crayfish biomass

As expected, recession rate and microtopography were less important for crayfish than fish, underscoring how particular hydrologic patterns could increase food availability for one species of top predator but not another [26]. Rather than dispersing to deep water before the dry-down, the Everglades crayfish, the most abundant species in our study, burrowed in place to avoid desiccation [77,78]. Thus, we were more likely to capture them in their preferred habitat, which typically has dense submerged aquatic vegetation. The positive association between crayfish biomass and density of submerged vegetation supported our hypothesis, and agrees with studies in the Everglades and other wetland systems. Crayfish select habitats with high structural complexity that provides protection from predators [38,79]. Moreover, crayfish can easily burrow through or under vegetation long after it has become too dense to allow movement by fish.

Dry-season crayfish biomass was also positively associated with water depth and negatively associated with days since dry-down. This was opposite to our hypothesized response and contrary to negative relationships between crayfish and water depth in sloughs and wet prairies in Blue Cypress Conservation area, Florida [38]. Our water depths were typically shallower than those of the earlier study, so crayfish at our sites were more likely to have already burrowed than those at sites with deeper water. Also, 60% of our identified crayfish were *P*. *alleni*, a species found most frequently at short- hydroperiod sites and well adapted to drying [78]. We may have seen a different pattern had our samples been dominated by *P*. *fallax*.

Little is known of the relationship between crayfish and the thickness of the floc layer. The short-hydroperiod regions inhabited by *P*. *alleni* were characterized by a thin floc layer, while the deep sloughs that accommodate *P*. *fallax* [78,80] have thick layers of floc. The strong support for wet-season crayfish biomass in the top models illustrates the first quantitative link between dry-season crayfish biomass and biomass of crayfish from the preceding wet season.

### Grass Shrimp Biomass

Less is known about grass shrimp behavior than fish or crayfish, but we predicted that they would respond like fish to the marsh drying because of their mobility and restriction to the water column [81]. Grass shrimp biomass increased with increasing rates of recession and increasing crayfish biomass, giving support to the idea that, like fish, grass shrimp form seasonal concentrations.

The positive association of grass shrimp with crayfish biomass is contrary to a previous hypothesis that crayfish affect habitat use and survival of shrimp [82]. Because crayfish burrow with drying, they do not form high concentrations in drying pools where they might affect shrimp density. Alternatively, shrimp may have responded to desiccation risk, rather than predation risk from crayfish in the drying pools, similar to our hypothesis for fish. Shrimp weakly responded, compared to fish, to the density of submerged vegetation, even though groups use vegetation as a refuge from aquatic predators [66,83,84]. Presence of vegetation, but not the density of vegetation, affects survival rate of shrimps [85], suggesting that our initial expectation of finding a similar response to that of fish may have been too simplistic.

## Conclusions

Strong support for the fish concentration \ habitat hypothesis coupled with the strong support for wet-season fish and crayfish biomass in the top models demonstrated that resource pulses are generated by the process of recession and microtopography transforming wet season prey production into prey concentrations. In wetland systems around the world, fish and crustaceans are a critical link in the trophic chain, providing food for top aquatic and terrestrial predators [86,87], but this link is not simply a positive function of prey abundance. Managing wetland ecosystems for prey production alone may increase the size and abundance of prey, but it would overlook key mechanisms that generate resource pulses by concentrating prey and making prey vulnerable to capture. These mechanisms may be especially critical in oligotrophic systems, where prey standing stocks are generally low [47].

Apex predators living in patchy environments, with unpredictable resources, may require periods of exceptionally high food availability to meet the elevated energetic demands of breeding [3,5]. These animals are typically mobile specialists that travel long distances to exploit spatially and temporally irregular food patches, and may be able to use resource pulses across a large spatiotemporal area [7,88]. Maintaining the critical mechanisms that control the timing, magnitude and location of resource availability is essential for natural ecosystem function and managing populations of apex predators.

## Supporting Information

S1 Appendix A schematic of the sampling components within a primary sampling unit. (PDF)

S2 Appendix Akaike's Information Criterion model selection for factors affecting fish, crayfish and grass shrimp biomass in the Florida Everglades, USA. (PDF)

S3 Appendix Justification for Variable Selection. (PDF)

S4 Appendix Manuscript data and metadata. (PDF)
