# Supplementary figures and images for: Mechanisms That Generate Resource Pulses in a Fluctuating Wetland
Source: PLoS One. 2016 Jul 22;11(7):e0158864. doi: 10.1371/journal.pone.0158864 (PMC4957811; doi:10.1371/journal.pone.0158864)

S1 Appendix. A schematic of the sampling components within a primary sampling unit.

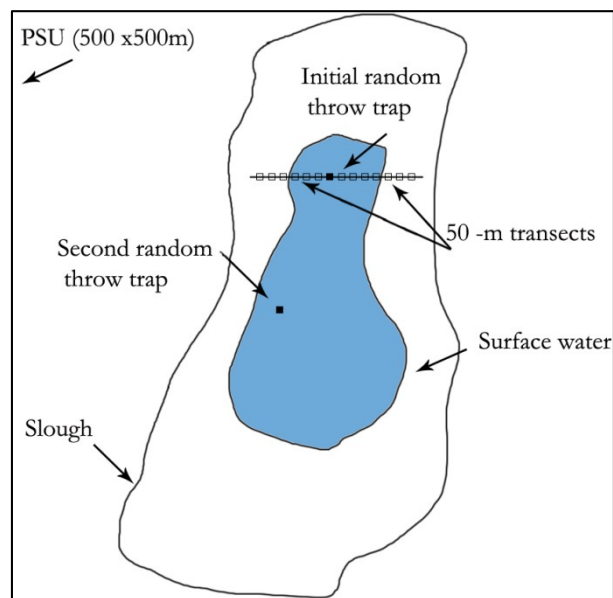

Supplement: S1 Appendix — (PDF) [file pone.0158864.s001.pdf]
