# Supplementary material for: Mechanisms That Generate Resource Pulses in a Fluctuating Wetland
Source: PLoS One. 2016 Jul 22;11(7):e0158864. doi: 10.1371/journal.pone.0158864 (PMC4957811; doi:10.1371/journal.pone.0158864)
Supplement: S2 Appendix — (PDF) [file pone.0158864.s002.pdf]

S2 Appendix. Akaike's Information Criterion model selection for factors affecting fish, crayfish and grass shrimp biomass in the Florida Everglades, USA.

Model	-2 LogLike	Parms	AICc	ΔAIC	w_i	R^2
Fish Models						
FISHBIOWET MCRIND REC SUBMGVEG FISHBIOWET*REC	1085.84	24	1137.17	0.00	0.75	0.3171
FISHBIOWET MCRIND REC SUBMGVEG	1090.94	23	1140.00	2.83	0.18	0.3073
GLOBAL	1083.73	27	1141.96	4.79	0.07	0.3211
FISHBIOWET REC MCRIND FISHBIOWET*REC	1108.57	23	1157.61	20.44	0.00	0.2723
FISHBIOWET REC MCRIND	1113.99	22	1160.76	23.59	0.00	0.2613
FISHBIOWET SUBMGVEG	1139.90	21	1184.50	47.29	0.00	0.2064
DSD REC MCRIND SUBMGVEG DSD*REC	1139.31	24	1190.49	53.32	0.00	0.2077
FLOC REC MCRIND SUBMGVEG	1143.11	23	1192.03	54.86	0.00	0.1994
DSD REC MCRIND SUBMGVEG	1143.13	23	1192.05	54.88	0.00	0.1994
FLOC REC MCRIND	1155.61	22	1202.28	65.11	0.00	0.1713
DSD REC MCRIND DSD*REC	1161.02	23	1209.92	72.75	0.00	0.1588
FISHBIOWET	1170.10	20	1212.30	75.18	0.00	0.1375
DSD REC MCRIND	1167.02	22	1213.67	76.50	0.00	0.1448
NULL	1224.11	19	1264.08	126.91	0.00	0.0000
Crayfish Models						
GLOBAL	972.52	27	1030.76	0.00	1.00	0.3824
FISHBIODRY DEPTH CRAYBIOWET SUBMGVEG	1023.27	23	1072.33	41.57	0.00	0.2878
FISHBIODRY REC DEPTH DSD FLOC	1050.74	24	1101.93	71.17	0.00	0.2311
CRAYBIOWET SUBMGVEG	1062.03	21	1106.58	75.82	0.00	0.2066
FISHBIODRY REC DEPTH FLOC	1067.11	23	1116.03	85.27	0.00	0.1954
CRAYBIOWET REC MCRIND	1080.25	22	1127.02	96.26	0.00	0.1656
FISHBIODRY REC DEPTH DSD	1085.92	23	1134.84	104.08	0.00	0.1524
FISHBIODRY REC DEPTH	1102.90	22	1149.57	118.81	0.00	0.1118
FISHBIODRY REC DSD	1116.88	22	1163.53	132.77	0.00	0.0769
NULL	1146.08	19	1186.05	155.29	0.00	0.0000
Shrimp Models						
GLOBAL	804.54	25	858.17	0.00	0.71	0.2758
CRAYBIODRY GRSHBIOWET REC MCRIND SUBMGVEG	810.86	23	859.92	1.75	0.29	0.2621
GRSHBIOWET MCRIND REC SUBMGVEG	834.27	22	881.07	22.90	0.00	0.2094
GRSHBIOWET REC MCRIND	839.55	21	884.07	25.90	0.00	0.1971
CRAYBIODRY DSD REC MCRIND SUBMGVEG	838.45	23	887.37	29.20	0.00	0.1997
CRAYBIODRY FLOC REC MCRIND SUBMGVEG	842.64	23	891.56	33.39	0.00	0.1898
CRAYBIODRY DSD SUBMGVEG	857.99	21	902.42	44.25	0.00	0.1527
CRAYBIODRY DSD SUBMGVEG FLOC	857.02	22	903.69	45.53	0.00	0.1551
CRAYBIODRY DSD	863.61	20	905.80	47.63	0.00	0.1387
REC MCRIND SUBMGVEG FLOC	866.10	22	912.77	54.61	0.00	0.1324
CRAYBIODRY SUBMGVEG	872.46	20	914.67	56.50	0.00	0.1163
NULL	915.39	18	953.16	95.00	0.00	0.0000
