# Supplementary material for: Mechanisms That Generate Resource Pulses in a Fluctuating Wetland
Source: PLoS One. 2016 Jul 22;11(7):e0158864. doi: 10.1371/journal.pone.0158864 (PMC4957811; doi:10.1371/journal.pone.0158864)
Supplement: S3 Appendix — (PDF) [file pone.0158864.s003.pdf]

S3 Appendix. Justification for Variable Selection

Microtopography is thought to be a fundamental contributor to concentrations of prey during the dry season (Jordan et al. 1998, Gawlik 2002, Trexler et al. 2002, Garrett 2007). Microtopographical relief not only produces shallow pools where prey are trapped and more available to wading birds, but it also creates refuges where prey that are not consumed can survive the seasonal dry-down and become a source for recolonization when the marsh is reflooded (Loftus et al. 1992, Kobza et al. 2003). Reduced sheet flow has caused a decrease in elevation difference between the ridge and the slough, resulting in a flattening of the ridge and slough landscape, decreased microtopographical variation and loss of distinctive ridge-slough vegetation patterns (Science Coordination Team 2003).

Vegetation has been shown to affect fish and macroinvertebrate community dynamics (Brazner and Beals 1997, Chick and McIvor 1997, Padial et al. 2009) and many studies have found that fish and macroinvertebrate densities are positively correlated with density of aquatic vegetation (Gilinsky 1984, Rozas and Odum 1988, Randall et al. 1996, Brazner and Beals 1997, Jordan et al. 1998, Hornung and Foote 2006, Warfe and Barmuta 2006).

There are at least two ways that flocculent matter could affect fish and macroinvertebrate communities. Floc assimilates phosphorus (Newman et al. 2001) and phosphorus enrichment has been shown to increase abundance of fish (Turner et al. 1999) and invertebrates (McCormick et al. 2004) in the Everglades. Floc is also a proxy for hydroperiod. Areas with longer hydroperiods tend to have a thicker floc layer, whereas areas with shorter hydroperiods have thinner floc. A longer hydroperiod equates to more time and space for growth and production of prey animals (Loftus and Eklund 1994).

Prolonged and uninterrupted periods of flooding provide prey with additional space for growth and reproduction, which increases the size and abundance of prey animals (Loftus and Eklund 1994). Days since dry-down has also been shown to affect the population dynamics of marsh fishes. Empirical studies (Jordan et al. 1998, Ruetz III. et al. 2005, Trexler et al. 2005) have shown that flagfish (*Jordanella floridae*) and marsh killifish (*Fundulus confluentus*) recover quickly following a dry-down, whereas bluefin killifish (*Lucania goodei*), least killifish (*Heterandria formosa*), and golden topminnow (*Fundulus chrysotus)* recover more slowly. Dry-down events can affect the community structure of large predator fishes (Chick et al. 2004), which may regulate small fish (Loftus and Eklund 1994, Dorn et al. 2006, Chick et al. 2008) and invertebrate (Dorn 2008, Liston et al. 2008) populations. The frequency of dry-down events

and extreme dry-down events in the study area are much more common now than historically (Fleming et al. 1994, Ogden 2005).

Recession of water levels during the dry season is one mechanism for concentrating aquatic wading bird prey in oligotrophic wetlands where standing stocks of prey are low. As the annual drying front moves through the landscape along a hydroperiod gradient, prey are trapped in microtopographical depressions along the trailing edge (DeAngelis 1994, Gawlik 2002). These high quality foraging patches are crucial to wading birds during their breeding season. Several studies have linked recession rates to the timing of wading bird colony formation and reproductive success (Kushlan 1979, Frederick and Collopy 1989, Herring et al. 2010). However, birds select particular recession rates based on the levels of prey biomass available in the landscape (Beerens et al. 2011).
